# Supplementary material for: High-Performance Dual-Ion Battery Based on Silicon–Graphene Composite Anode and Expanded Graphite Cathode
Source: Molecules. 2023 May 23;28(11):4280. doi: 10.3390/molecules28114280 (PMC10254290; doi:10.3390/molecules28114280)
Supplement: Supplementary file 1 [file molecules-28-04280-s001.zip › molecules-2387473-supplementary.pdf]

Supplementary Materials

High-Performance Dual-Ion Battery Based on Silicon–Graphene Composite Anode and Expanded Graphite Cathode

Guoshun Liu †, Xuhui Liu †, Xingdong Ma, Xiaoqi Tang, Xiaobin Zhang, Jianxia Dong, Yunfei Ma, Xiaobei Zang, Ning Cao and Qingguo Shao *

School of Materials Science and Engineering, China University of Petroleum (East China), Qingdao 266580, China

* Corresponding author. E-mail addresses: qgshao@upc.edu.cn

† These authors contributed equally to this work.

Table of Contents

1. XRD and Raman plot of graphite and EG

2. GCD curves and rate diagrams for graphite and EG

3. GCD curves at different current densities

4. GCD curves of $LiFePO_4$//Si@G-5 LIB at different current densities

5. Cycle curve of $LiFePO_4$//Si@G-5 LIB

6. Calculation of Li^+ diffusivity

7. Comparison of energy density and power density of EG//Si@G-5 DIB and $LiFePO_4$//Si@G-5 LIB at different current densities

8. Electrochemical performance calculation

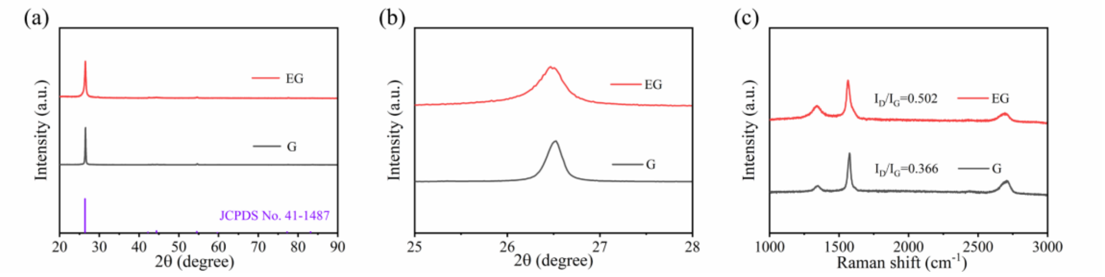

Figure S1. (a-b) XRD comparison of graphite and EG, **(c)** Raman plot comparison of graphite and EG.

The XRD of graphite and EG are shown in Figure S1a, b. The characteristic diffraction angles of graphite and EG are 26.53 and 26.47°, respectively, indicating that EG still retains the physicochemical properties of graphite and possess a larger interlayer distance. Figure S1c shows the Raman plots of both, and it can be observed that the peak intensity of EG at 1347 and 1576 cm^{-1} are higher than graphite. The I_D/I_G of EG is also higher, which indicates an increase in defects and layer spacing of EG.

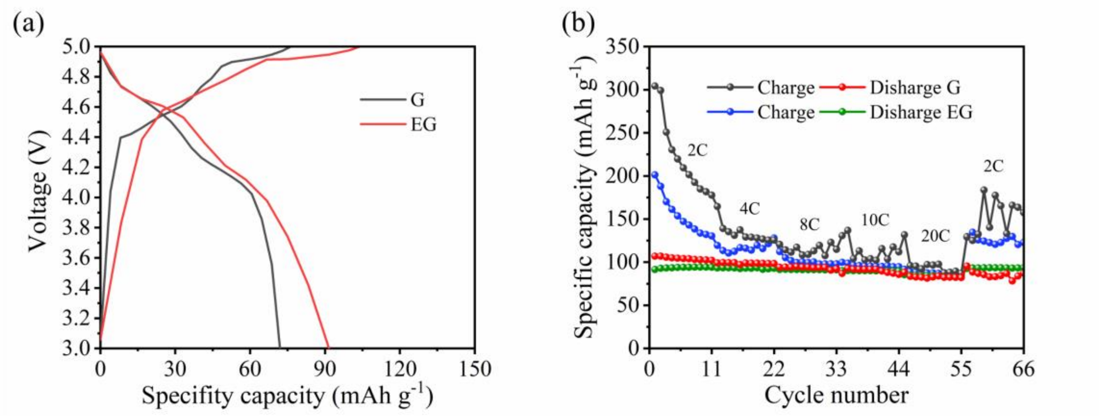

Figure S2. (a) GCD curves of graphite and EG at 10 C, **(b)** Comparison of rate performance between graphite and EG.

Figure S2a shows the GCD curves of graphite and EG at 10 C. The discharge specific capacities of graphite and EG are 71.98 and 91.59 mAh g^{-1}, respectively, which are attributed to the larger layer spacing. The rate performance is shown in Figure S2b. After experiencing cycles at different current densities, the capacity of graphite fluctuates considerably, which may be due to the kinetic conditions for Li^+ diffusion in graphite is relatively poor at high rates. The specific discharge capacity of EG at 2, 4, 8, 10 and 20 C is 93.22, 92.62, 89.15, 87.47 and 83.50 mAh g^{-1}, respectively. When it returns to 2 C, the specific discharge capacity returns to 92.62 mAh g^{-1}. In comparison, the overall stability of EG is better, which represents a better cycle reversibility of EG.

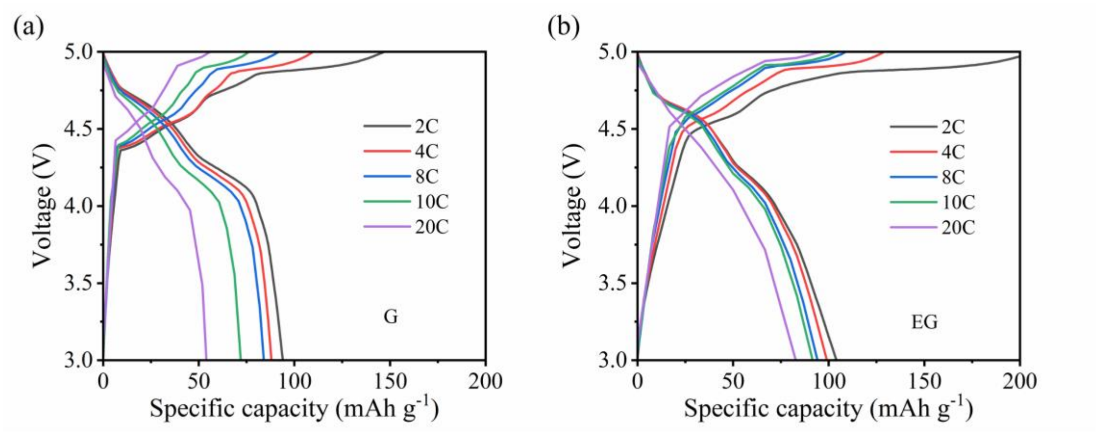

Figure S3. GCD curves at different current densities: **(a)** graphite, **(b)** EG.

Figure S3 shows the GCD curves of graphite and EG at different current densities. The insertion/extraction of the anion is mainly divided into three stages and shows similar plateaus and trends, but the long-term cycle stability is poor. Notably, the charging plateau of EG is reduced to two and smoother, which represents a very stable process of anion insertion. This results demonstrate EG shows fast intercalation kinetic to accommodate anions.

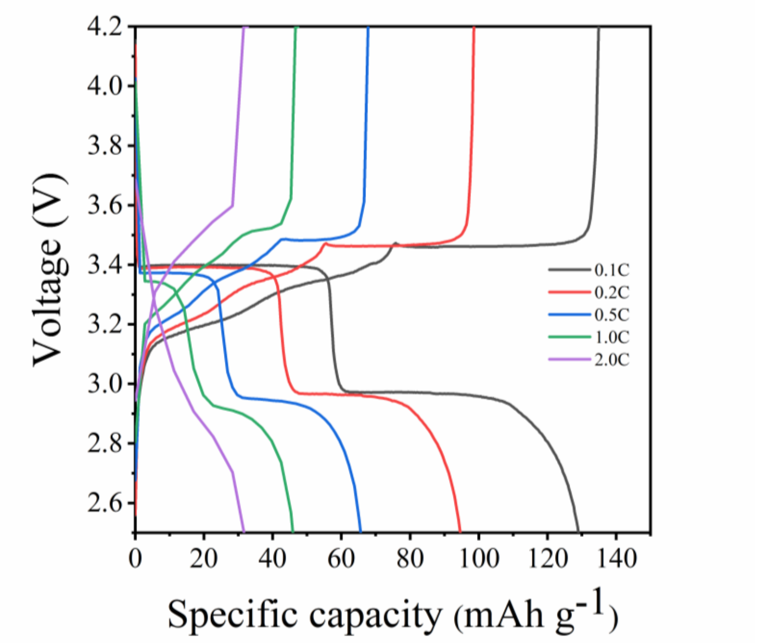

Figure S4. GCD curves of $LiFePO_4$//Si@G-5 LIB at different current densities.

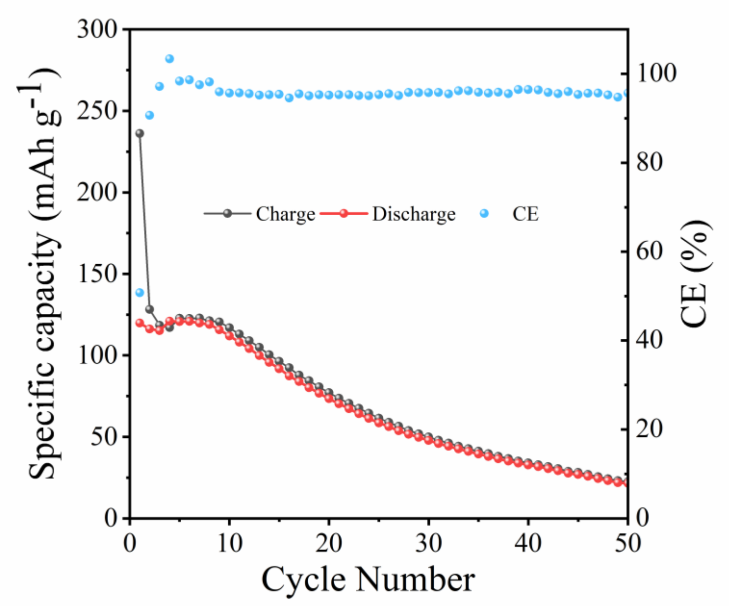

Figure S5. 50 cycles performance of $LiFePO_4$//Si@G-5 LIB at 0.5 C.

Calculation of Li+ diffusivity

Equation S1: The sloping line in the low frequency region of the EIS curve is related to the Warburg impedance, and the diffusion coefficient of lithium ions can be calculated using the following equation.

$$D_{Li^+} = \frac{R^2T^2}{2A^2n^4F^4C^2\sigma^2}$$

Where, D_{Li+} represents the diffusion coefficient of Li^+. R is the gas constant, T is the absolute temperature, A is the area of the electrode, F is the Faraday constant, n is the number of electrons in the electrochemical reaction, C is the concentration of Li+, and σ is the Warburg coefficient. Note that σ can be obtained from the slope of Z' and $\omega^{-1/2}$, and the linear relationship between Z' and ω is shown by the equation

$$Z' = R + \sigma\omega^{-1/2}$$

$$\omega = 2\pi f$$

The fitted curves were calculated as σ_0 = 170.51, σ_{50} = 669.40, and σ_{200} = 1328.49. Resulting in Li^+ diffusion coefficients of 1.37×10^{-21}, 8.90×10^{-23}, and 2.26×10^{-23} $cm^2\ s^{-1}$ for the three, respectively.

Comparison of energy density and power density of EG//Si@G-5 DIB and LiFePO4//Si@G-5 LIB at different current densities

Table S1. Energy density and power density of EG//Si@G-5 DIB at different current densities.

Current Density (C)	2	4	8	10	20
Cathode capacity (mAh g^{-1})	86.97	80.15	55.88	46.44	41.94
Cell capacity (mAh g^{-1})	83.41	76.87	53.59	44.54	40.22
Energy Density (Wh kg^{-1})	367.84	332.8	196.68	158.11	138.37
Power Density (W kg^{-1})	855.43	1664.2	2185.3	2635.21	3459.18

Table S2. Energy density and power density of $LiFePO_4$//Si@G-5 LIB at different current densities.

Current Density (C)	0.1	0.2	0.5	1	2
Cell capacity (mAh g^{-1})	129.45	95.64	66.32	46.25	31.93
Energy Density (Wh kg^{-1})	386.76	284.07	195.73	135.56	93.58
Power Density (W kg^{-1})	50.89	102.18	257.54	502.07	1039.78

Electrochemical performance calculation

In order to achieve the best performance of the battery, the cathode and anode materials need to be matched. Control the N/P ratio to 1.1 (N/P = anode active material gram capacity × anode surface density × anode active material content ratio / (cathode active material gram capacity × cathode surface density × cathode active material content ratio)).

The mass is based on the mass of the full cell (Sum of active materials and electrolyte). Specific capacity ($C_{cathode}$ (based on EG mass in cathode)) and discharge medium voltage (V_m) obtained from GCD curves at different current densities.

Equation S2:

The capacity of the full cell is calculated according to: $C_{cell}=C_{cathode} \times m_{cathode}/(m_{cathode}+m_{anode}+m_{electrolyte})$

The energy density of the full cell is calculated according to: $E_{cell}=C_{cell} \times V_m$

The power density of the full cell is calculated according to: $P_{cell}=E_{cell}/t$
